# UAV Trajectory Design and Power Optimization for Terahertz Band-Integrated Sensing and Communications

**DOI:** 10.3390/s23063005

**Published:** 2023-03-10

**Authors:** Ying Gao, Hongmei Xue, Long Zhang, Enchang Sun

**Affiliations:** 1 School of Information and Electrical Engineering, Hebei University of Engineering, Handan 056038, China; 2 Chongqing Engineering Research Center of Intelligent Sensing Technology and Microsystem, Chongqing 400065, China; 3 Beijing Advanced Innovation Center for Future Internet Technology, Beijing University of Technology, Beijing 100124, China; 4 Faculty of Information Technology, Beijing University of Technology, Beijing 100124, China

**Keywords:** terahertz band, UAV, integrated sensing and communications, power optimization, trajectory design

## Abstract

Sixth generation (6G) wireless networks require very low latency and an ultra-high data rate, which have become the main challenges for future wireless communications. To effectively balance the requirements of 6G and the extreme shortage of capacity within the existing wireless networks, sensing-assisted communications in the terahertz (THz) band with unmanned aerial vehicles (UAVs) is proposed. In this scenario, the THz-UAV acts as an aerial base station to provide information on users and sensing signals and detect the THz channel to assist UAV communication. However, communication and sensing signals that use the same resources can cause interference with each other. Therefore, we research a cooperative method of co-existence between sensing and communication signals in the same frequency and time allocation to reduce the interference. We then formulate an optimization problem to minimize the total delay by jointly optimizing the UAV trajectory, frequency association, and transmission power of each user. The resulting problem is a non-convex and mixed integer optimization problem, which is challenging to solve. By resorting to the Lagrange multiplier and proximal policy optimization (PPO) method, we propose an overall alternating optimization algorithm to solve this problem in an iterative way. Specifically, given the UAV location and frequency, the sub-problem of the sensing and communication transmission powers is transformed into a convex problem, which is solved by the Lagrange multiplier method. Second, in each iteration, for given sensing and communication transmission powers, we relax the discrete variable to a continuous variable and use the PPO algorithm to tackle the sub-problem of joint optimization of the UAV location and frequency. The results show that the proposed algorithm reduces the delay and improves the transmission rate when compared with the conventional greedy algorithm.

## 1. Introduction

Following the birth of various emerging applications, such as holographic communication, sensory interconnection, three-dimension immersive experiences, and the metaverse, terahertz (THz) band communication is envisioned as one of the key enabling technologies to satisfy the needs of emerging applications [1,2]. Specifically, the ultra-wide THz band that ranges from 0.1 THz to 10 THz promises to support applications with a high quality of service and terabits per second data rates [3]. The THz frequency band will provide new applications for future ultra-high data rate communication because of the ultra-wide THz band [4]. In addition to communication applications, the THz frequency will also enable high resolution and accuracy sensing, such as radar, augmented human senses, and other scenarios [5]. Furthermore, THz networks can realize massive communication connectivity with plenty of available spectrum resources, as more than 10 billion devices are expected to be connected in the coming years [6].

However, the realization of ultra-bandwidth terahertz communication faces three major technical challenges: the first is to technically reach a high-speed terahertz signal of over 100 Gbps, the second is to be able to process high-speed terahertz signals in real time, and the third is to overcome the high channel loss characteristics of terahertz signals. We are going to focus on the third case.

On the one hand, the THz frequency has the characteristic of a high path loss; thus, with the increase in carrier frequencies and communication distances, THz wave propagation suffers from higher spreading losses and stronger non-line-of-sight path losses attributed to scattering, reflection, diffraction, and shadowing [7]. Thus, non-line-of-sight transmission in the THz spectrum is rarely received at the receiving end due to these phenomena. However, line-of-sight transmission is almost nonexistent because of the amount of cover in cities. The deployment of UAVs has been regarded as a complementary alternative to existing cellular systems to achieve higher transmission efficiency and capacity [8]. Therefore, UAVs are needed as aerial base stations to provide line-of-sight transmission links for THz frequencies.

On the other hand, THz communications are highly affected by molecular absorption loss caused by water molecules in the atmosphere. The atmospheric water molecule content varies during the day, and traditionally, the relevant papers, such as [9], usually just give a constant value, which causes errors when choosing channels. Error deviation from the traditional estimation method of path loss is unacceptable in the THz frequency. However, THz-UAVs can detect real-time environmental changes through sensing signals, thus measuring terahertz channel parameters. Therefore, the performance of sensing systems should be taken into account when optimizing THz communication resources [10,11]. In short, integrated sensing and communications endows THz-UAV communication networks with new abilities to interact to perceive the physical world and then improve user information rates. Thus, this topic has importance. We included Table 1 to clearly demonstrate the novelty of our paper and this will be discussed in Section 2. Thus, it is highly necessary to achieve integrated sensing and communications for THz transmission.

**Figure 1:**
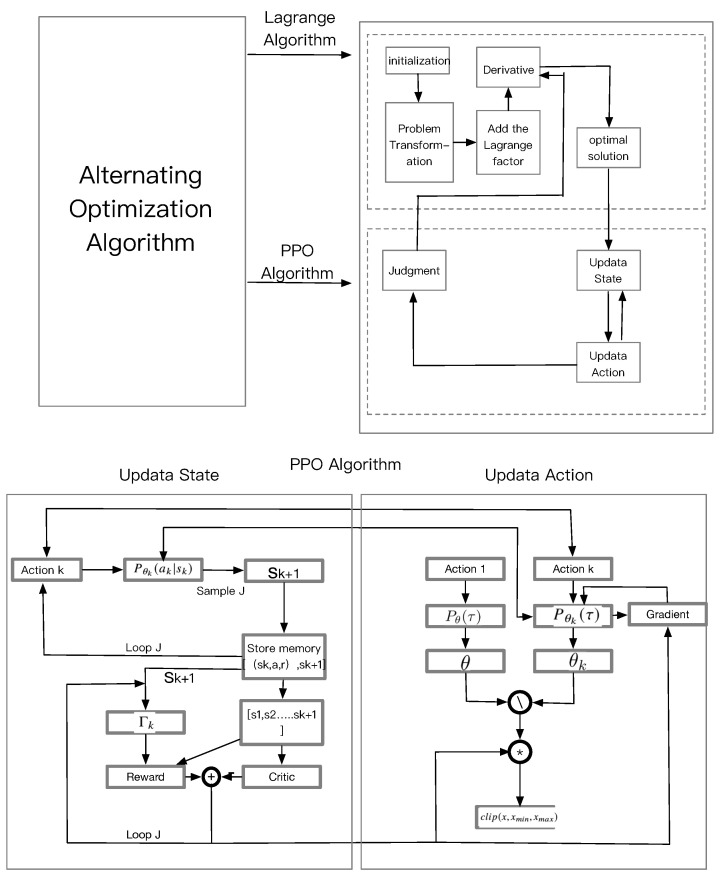
Alternating optimization algorithm.

Against this background, in this paper, we propose a THz band sensing-assisted UAV communication network to provide wireless communication for users. Particularly, we focus on downlink communications while jointly optimizing the UAV trajectory, frequency association, and power association. The main contributions of our work include:

• Designing a new sensing and communication power optimization method that considers interference between sensing and communication signals in a THz sensing-assisted UAV communication network.
• Formulating an optimization problem (Figure 1) and proposing an efficient alternative optimization to solve this problem. First, we use the Lagrangian dual decomposition method to obtain the power of sensing and communication with a fixed trajectory. Second, we use the policy optimization (PPO) algorithm for joint optimization of the UAV location and frequency association with a fixed power of sensing and communication.
• Designing a PPO algorithm for optimizing the UAV trajectory and frequency association. The PPO algorithm uses the critic network with global information and the actor network with local information to achieve cooperation to explore the angle of UAV and the frequency association.

The rest of this paper is organized as follows: The prior works are described in Section 2. In Section 3, the system model is described. In Section 4, the decomposition problem and the joint optimization design are presented. In Section 5, the simulation results are provided and discussed. Finally, this paper is concluded in Section 6.

## 2. Prior Works

The study of UAVs is considered as a new frontier field [12,13,14,15,16]. In [12], the authors designed an optimization problem to maximize the sum rate of a satellite and aerial integrated network. In [13], the authors aimed to maximize the energy efficiency of UAV-enabled communication by optimizing its trajectory. In [14], the authors designed a limited storage space and energy for a UAV-assisted wireless communication system to realize the multi-user communication. In [15], the authors proposed a new protocol for UAV-to-UAV and UAV-to-GCS communication. In [16], the authors give a short overview of the possible threats, attacks, and countermeasures related to UAV communications.

To exploit THz band UAV wireless communication, some initial works have considered THz-enabled aerial communications [17,18,19]. In [17], the authors proposed a UAV-to-user THz sub-band association scheme to eliminate interference in the THz frequency transmission. They proved that terahertz frequencies could be used for communication, and extensions of the wireless charging window and THz-transmitting window are derived. In [18], the authors minimized the total delays of the uplink and downlink transmissions between the UAV and the users by jointly optimizing the location of the operating UAV and the bandwidth of the users, as well as minimizing the transmitting power of the users. They optimized the performance of the drones to communicate using terahertz frequencies. In [19], the authors studied how UAVs support THz communications and an IRS was deployed to help the transmission. Yijin Pan’s aim is to maximize the minimum average rates of all users. They optimized and evaluated the resource optimization problem for terahertz UAVs.

Many works have been dedicated to integrated sensing and communications [20,21,22]. In [20], the authors provided a brief explanation of communication rate maximization theory. Their goals were to research the basic communications phenomenology and to study dealing with systems in an information theory context. In [21], the authors aimed to further investigate the achievable performance of spectrally overlapping radar and communication systems by conjugating the detection. In [22], the authors developed a new approach for producing joint radar communications performance bounds. The authors studied the boundary question of combined communication and sensing.

There are growing research interests in power optimization [23,24,25,26]. In [23], the authors’ design objective was to minimize the total transmission power of both the satellite and BS with a limited onboard power resource. In [24], the authors designed an objective function to maximize the system secrecy energy efficiency under the constraint of the total transmission power budget. In [25], the authors investigated the energy minimization problem of a UAV-assisted data collection sensor network. In [26], the authors designed a function that maximized the sum rate in a satellite–terrestrial integrated network, aiming to satisfy the constraints of per-antenna transmission power and quality-of-service requirements of both satellite and cellular users.

Although there are many papers on UAV communications, most of these existing works [20,21,22,23,24,25,26] do not focus on integrated sensing and communication. Therefore, this area is well worth studying.

We have summarized the relevant work in Table 1.

## 3. System Model and Problem Formulation

### 3.1. System Model

**Figure 2:**
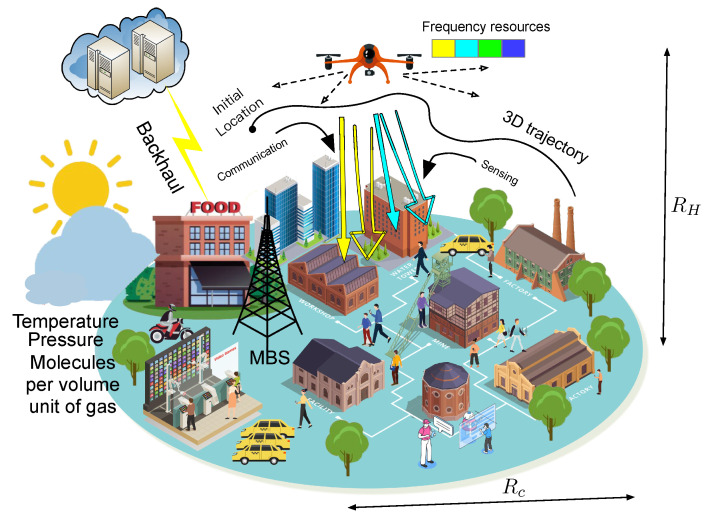
Illustration of a terahertz band integrated sensing and communications network.

Let us now consider a downlink from a THz UAV to *N* users during time horizon *T*, shown in Figure 2. We suppose that the user equipment is taken as a two-dimensional (2D) homogeneous Poisson point process (PPP) $\Phi_{u}$ with intensity $\lambda_{u}$. For ease of calculation, the time horizon of *T* is equally divided into K+1 time slots with length $\frac{T}{K+1}$. THz-UAVs use integrated sensing and communication to improve the performance of system. As a result of shared spectrum resources in sensing and communication signals, it is challenging to achieve the critical trade-off between these two integrated functionalities. In order to reduce the interference of communication and sensing signals of the same frequency, at time slot 0, the UAV sends sensing signals and users receive sensing signals. During the time slot of 1 to K+1, the UAV sends communication and sensing signals and users receive communication and sensing signals.

Therefore, for *N* targets, the user signal received at time slot *k* can be expressed as:

(1) $$z_{k}=\sum_{n=1}^{N}z_{k}^{n}=\sum_{n=1}^{N}\left[h_{k}^{n}{\left(P_{k}^{S,n}\right)}^{\frac{1}{2}}S_{k}+h_{k}^{n}{\left(P_{k}^{C,n}\right)}^{\frac{1}{2}}C_{k}\right]+n_{k},$$

where $S_{k}$ is the sensing signal, $C_{k}$ is the communication signal, $P_{k}^{S,n}$ and $P_{k}^{C,n}$ are the transmitting power of sensing and communication signals at time slot *k*, respecitvely, and $h_{k}^{n}$ is the THz channel gain from the UAV to the user *n*.

Without loss of generality, we assume that the UAV is moving with a constant speed denoted by *V*, and the location of the UAV is denoted by $L_{k}=\left(x_{k},y_{k},H\right)$ at time slot *k*. Here, the altitude, *H*, of the UAV is assumed to be constant. Therefore, the following coordinates of the UAV at time slot *k* should be satisfied

(2) $$\begin{array}{cc} x_{k} & =x_{k-1}+Vkcos\psi_{k-1}^{n},\\ y_{k} & =y_{k-1}+Vksin\psi_{k-1}^{n}, \end{array}$$

where $\psi_{k-1}^{n}\in \left[0,4\pi \right]$ is the direction of the UAV at time slot k−1 from the UAV to the user *n*.

The following trajectory constraints of the UAV should be satisfied [27]

(3) $$\begin{array}{cc} \; & {\left(L_{1}-L_{0}\right)}^{2}\leq {\left(V\frac{T}{K+1}\right)}^{2},\\ \; & {\left(L_{k}-L_{k-1}\right)}^{2}\leq {\left(V\frac{T}{K+1}\right)}^{2},\\ \; & {\left(L_{K}-L_{K-1}\right)}^{2}\leq {\left(V\frac{T}{K+1}\right)}^{2}, \end{array}$$

where $L_{0}$ and $L_{K}$ are the initial location and finial location, respectively.

Considering the LoS transmission, the path loss between the UAV and the user, *n*, can be written as [28]:

(4) $$h_{k}^{n}\left(f_{k,i}^{n},\varepsilon_{n}\left(f_{k,i}^{n},\epsilon_{k}\right)\right)=H_{k}^{Spr}\left(f_{k}^{n}\right)H_{k}^{Abs}\left(f_{k}^{n},\varepsilon_{n}\left(f_{k,i}^{n},\epsilon_{k}\right)\right)e^{-j2\pi f_{k}^{n}},$$

where $f_{k,i}^{n},i\in \left\{f_{1},f_{2},...,f_{I}\right\}$ is the carrier frequency adpoted by the UAV for communicating with user *n* and $\varepsilon_{n}\left(f_{k,i}^{n},\epsilon_{k}\right)$ is the absorption coefficient parameter related to the carrier frequency $f_{k,i}^{n}$ and the number of water molecules in the atmosphere, $\epsilon_{k}$, at time slot *k*.

The free space direct ray or LoS channel transfer function, $H_{LoS}$, consists of the spreading loss function, $H_{Spr}$, and the molecular absorption loss function, $H_{Abs}$. The transfer function due to the spreading loss is given by:

(5) $$H_{k}^{Spr}\left(f_{k,i}^{n}\right)=\frac{c}{4\pi f_{k,i}^{n}d_{k}^{u,n}}.$$

The transfer function of the molecular absorption loss can be expressed as:

(6) $$H_{Abs}\left(f_{k,i}^{n},\varepsilon_{n}\left(f_{k,i}^{n},\epsilon_{k}\right)\right)=e^{-\varepsilon_{n}\left(f_{k,i}^{n},\epsilon_{k}\right)d_{k}^{u,n}},$$

where the accuracy of $\varepsilon_{n}\left(f_{k,i}^{n},\epsilon_{k}\right)$ is positively correlated with the sensing power. For the specific formula, please refer to [22].

The environmental parameters change slowly; therefore, we can use time slot k−1 to represent the sensing estimate value at time slot *k*. The communication signal of the user at time slot *k* is the total signal received at time slot *k*, $z_{k}^{n}$, minus the sensing estimated signal at time slot *k*. Thus, user *n* receives communication signals at time slot *k*, which can be determined by:

(7) $$C_{k}^{n}=z_{k}^{n}-{\tilde{h}}_{k-1}^{n}\left(f_{k-1,i}^{n},\varepsilon_{n}\left(f_{k-1,i}^{n},\epsilon_{k-1}\right)\right){\left(P_{k-1}^{S,n}\right)}^{\frac{1}{2}}S_{k-1},$$

where ${\tilde{h}}_{k-1}^{n}\left(f_{k-1,i}^{n},\varepsilon_{n}\left(f_{k-1,i}^{n},\epsilon_{k-1}\right)\right)$ is the THz channel gain at frequency $f_{k,i}^{n}$, which is obtained by sensing signals.

The THz-UAV needs to extract sensing signals to estimate $\varepsilon_{n}\left(f_{k,i}^{n},\epsilon_{k}\right)$ and to assign a THz carrier to users. The accuracy of $\varepsilon_{n}\left(f_{k,i}^{n},\epsilon_{k}\right)$ affects the THz carrier distribution. Similarly, at time slot *k*, the sensing signals received by the THz-UAV can be expressed as:

(8) $$S_{k}^{n}=z_{k}^{n}-{\tilde{h}}_{k-1}^{n}\left(f_{k-1,i}^{n},\varepsilon_{n}\left(f_{k-1,i}^{n},\epsilon_{k-1}\right)\right){\left(P_{k}^{C,n}\right)}^{\frac{1}{2}}C_{k}.$$

As a result of sensing and communication signals sharing spectrum resources, the error between the real sensing signal at time *k* and the estimated sensing signal will interfere with communication signals. In addition, other users using the same THz carrier will also interfere with user *n*. Therefore, the SINR received at user *n* can be expressed as:

(9) $$\gamma_{k}^{n}=\frac{p_{k}^{C,n}{\tilde{h}}_{k-1}^{n}\left(\cdot \right)}{N_{0}+p_{k}^{S,n}{\tilde{h}}_{k-1}^{n}\left(\cdot \right)-p_{k-1}^{S,n}{\tilde{h}}_{k-1}^{n}\left(\cdot \right)+\sum_{j=1}^{j/n}\left(p_{k}^{C,j}{\tilde{h}}_{k-1}^{j}\left(\cdot \right)+p_{k}^{S,j}{\tilde{h}}_{k-1}^{j}\left(\cdot \right)-p_{k-1}^{S,j}{\tilde{h}}_{k-1}^{j}\left(\cdot \right)\right)},$$

where $N_{0}$ is the additive white gaussian noise power at user *n* using the *i*th carrier frequency of the THz band.

Correspondingly, the achievable downlink rate of the UAV to user *n* can be written as [29]:

(10) $$r_{k}^{n}=Blog\left(1+\gamma_{k}^{n}\right),$$

where *B* is the bandwidth of the UAV to user *n*, which is assumed to be equal for each user.

Thus, the delay of all the users at time slot *k* can be written as follows:

(11) $$\Phi_{k}=\sum_{n=1}^{N}\frac{D_{n}}{B_{u}log\left(1+\gamma_{k}^{n}\right)},$$

where $D_{n}$ is the amount of data required by user *n*.

### 3.2. Problem Formulation

Using the above setup, we aim to minimize the delay over time slots K+1 by jointly optimizing the UAV trajectory, frequency association, and transmission power. This optimization problem is mathematically formulated as:

(12) $$\min_{f_{k}^{n},L_{k},p_{k}^{C,n},p_{k}^{S,n}}\sum_{k=1}^{K}\sum_{n=1}^{N}\frac{D_{n}}{B_{u}log\left(1+\gamma_{k}^{n}\right)}$$

so that

$$\begin{array}{cc} C1: & \sum_{i=1}^{I}f_{k,i}^{n}\leq 1,\\ C2: & {\left(L_{1}-L_{0}\right)}^{2}\leq {\left(V\frac{T}{K}\right)}^{2},\\ C3: & {\left(L_{k}-L_{k-1}\right)}^{2}\leq {\left(V\frac{T}{K}\right)}^{2},\\ C4: & {\left(L_{K}-L_{k-1}\right)}^{2}\leq {\left(V\frac{T}{K}\right)}^{2},\\ C5: & \sum_{n=1}^{N}p_{k}^{C,n}+p_{k}^{S,n}\leq P_{k}^{max}, \end{array}$$

where constraint C1 ensures each user can be associated with one carrier frequency at each time slot *k*. C2–C4 ensure that the UAV cannot exceed the maximum speed at the time horizon *T*. C5 limits the maximum transmission power of sensing signals and communication signals.

## 4. Problem Decomposition and Joint Optimizing Design

### 4.1. Problem Decomposition

We note that the challenges of solving problem (12) lie in the following reasons. First, the optimization variable $f_{k,i}^{n}$ for user *n* at time slot *k* is binary, and thereby the feasible set of problem (12) is non-convex. Second, the variables $L_{k}$ and $f_{k,i}^{n}$ are strongly coupled with the sensing power and communication power. Hence, problem (12) is a mixed integer non-convex optimization problem and in general there is no standard method for solving it efficiently.

To tackle the above challenges, we decompose the original problem (12) into two sub-problems by separating the power allocation optimization (P1) and the trajectory and frequency variables (P2).

We first consider the power variables $p_{k}^{C,n}$ and $p_{k}^{S,n}$ in (P1) by fixing the trajectory variable $L_{k}^{n}$ and the frequency variable $f_{k,i}^{n}$. Therefore, subproblem (P1) can be expressed as:

(13) $$\begin{array}{cc} \left(P1\right): & \min_{p_{k}^{C,n},p_{k}^{S,n}}\frac{D_{n}}{B_{u}log\left(1+\gamma_{k}^{n}\right)}\\ \; & s.t.\;C5 \end{array}$$

We next consider the trajectory variable in (14) by fixing the UAV power allocation variables $p_{k}^{C,n}$ and $p_{k}^{S,n}$. Therefore, subproblem (P2) can be formulated by:

(14) $$\begin{array}{cc} \left(P2\right): & \min_{L_{k},f_{k,i}^{n}}\sum_{n=1}^{N}\frac{D_{n}}{B_{u}log\left(1+\gamma_{k}^{n}\right)}\\ \; & s.t.\;C1-C4 \end{array}$$

The two subproblems are separately optimized with multiple iterations. In the j+1-th iteration $\left(j=0,1,2,\cdot \cdot \cdot ,j_{max}\right)$, we first optimize $p_{k}^{C,n}$ and $p_{k}^{S,n}$ using the Lagrange multiplier method in (P1) with fixed trajectory variable $L_{k}^{n}$ and frequency variable $f_{k,i}^{n}$, and find that the solution can be expressed by $p_{k}^{*C,n},p_{k}^{*S,n}$. We then optimize the variables $L_{k}$ and $f_{k,i}^{n}$ in (P2) using the PPO algorithm, and find that the solution can be expressed by $L_{k}^{j+1},f_{k,i}^{n,j}$. After the solution converges or a the maximum number of iterations or $j_{max}$ is reached, the solution of (14) can be obtained.

### 4.2. Joint Optimization Design

In this section, we will present the solution to the above two subproblems, and then propose a joint algorithm via separately optimizing the subproblems in an iterative way.

#### 4.2.1. Joint Sensing and Communication Power

Before solving (12), we first demonstrate the convexity of this problem in Theorem 1 shown below.

**Theorem** **1.** *Problem (P1) is convex. Please refer to Appendix A.*

As a result of sub-problem (13) being a convex problem, we chose the Lagrangian dual decomposition method to solve it and obtain the optimal solution of $p_{k}^{*S,n}$ and $p_{k}^{*C,n}$. The Lagrangian function of (P1) can be given by:

(15) $$L\left(p_{k}^{S,n},p_{k}^{C,n},f_{k,i}^{n},\chi ,\eta ,\vartheta \right)=\Phi^{1}+\sum_{k=1}^{K}\eta_{k}\left(\sum_{n=1}^{N}p_{k}^{C,n}+p_{k}^{S,n}-P^{max}\right),$$

where $\eta_{k}$ is the Lagrange multiplier associated with constraint C5.

Since (P1) is convex, it satisfies the Karush–Kuhn–Tucker (KKT) conditions, which can be specifically derived as:

(16) $$\eta_{k}\left(\sum_{n=1}^{N}p_{k}^{*C,n}+p_{k}^{*S,n}-P_{k}^{max}\right)=0,$$

(17) $$\frac{\partial L(\cdot \cdot \cdot )}{\partial p_{k}^{C,n}}=-\frac{B_{u}}{ln2}\frac{1}{log(1+r_{k}^{n})}\frac{1}{1+r_{k}^{n}}\lambda_{1}+\sum_{k=1}^{K}\eta_{k}=0,$$

(18) $$\begin{array}{cc} \frac{\partial L(\cdot \cdot \cdot )}{\partial p_{k}^{S,n}}= & \frac{B_{u}}{ln2}\sum_{k=1}^{K}\frac{p_{k}^{C,n}{\tilde{h}}_{k-1}^{n}\left(\cdot \right)+N_{0}+p_{k}^{S,n}{\tilde{h}}_{k-1}^{n}\left(\cdot \right)-p_{k-1}^{S,n}{\tilde{h}}_{k-1}^{n}\left(\cdot \right)+\sum_{j=1}^{j/n}p_{k}^{C,j}{\tilde{h}}_{k-1}^{j}\left(\cdot \right)+p_{k}^{S,j}{\tilde{h}}_{k-1}^{j}\left(\cdot \right)-p_{k-1}^{S,j}{\tilde{h}}_{k-1}^{j}\left(\cdot \right)}{{\left(N_{0}+p_{k}^{S,n}{\tilde{h}}_{k-1}^{n}\left(\cdot \right)-p_{k-1}^{S,n}{\tilde{h}}_{k-1}^{n}\left(\cdot \right)+\sum_{j=1}^{j/n}p_{k}^{C,j}{\tilde{h}}_{k-1}^{j}\left(\cdot \right)+p_{k}^{S,j}{\tilde{h}}_{k-1}^{j}\left(\cdot \right)-p_{k-1}^{S,j}{\tilde{h}}_{k-1}^{j}\left(\cdot \right)\right)}^{2}}\\ \; & \times \frac{1}{log(1+r_{k}^{n})}+\sum_{k=1}^{K}\eta_{k}=0. \end{array}$$

Case 1. If $\eta_{k}\neq 0$, the KKT conditions (16) can be written as:

(19) $$\eta_{k}\left(\sum_{n=1}^{N}P_{k}^{S,max}+P_{k}^{C,max}-p_{k}^{C,n}-p_{k}^{C,n}\right)=0,$$

where $P_{k}^{C,max}$ and $P_{k}^{S,max}$ indicate the maximum sensing and communication powers for time slot *k*, respectively. $\eta_{k}\neq 0$; therefore, the solution of ${\overset{´}{p}}_{k}^{S,n}$ and ${\overset{´}{p}}_{k}^{S,n}$ in (P1) can be denoted in closed-form as ${\overset{´}{p}}_{k}^{C,n}=P_{k}^{C,max}$ and ${\overset{´}{p}}_{k}^{S,n}=P_{k}^{S,max}$.

Case 2. If $\eta_{k}=0$, combining $\eta_{k}=0$ and (17) and (18), the solution of ${\overset{´}{p}}_{k}^{S,n}$ and ${\overset{´}{p}}_{k}^{S,n}$ in (P1) can be denoted in closed-form as:

(20) $${\overset{‘}{p}}_{k}^{S,n}=\frac{\sum_{j=1}^{j/n}p_{k}^{C,j}h_{k}^{j}\left(f_{k,i}^{j}\right)}{h_{k}^{n}\left(f_{k,i}^{j}\right)},$$

(21) $${\overset{‘}{p}}_{k}^{C,n}=\frac{-p_{k-1}^{S,n}{\tilde{h}}_{k-1}^{n}\left(\cdot \right)+\sum_{j=1}^{j/n}p_{k}^{C,j}.{\tilde{h}}_{k-1}^{j}\left(\cdot \right)+p_{k}^{S,j}{\tilde{h}}_{k-1}^{j}\left(\cdot \right)-p_{k-1}^{S,j}{\tilde{h}}_{k-1}^{j}\left(\cdot \right)}{{\tilde{h}}_{k-1}^{n}}.$$

In summary, the optimal solutions of ${\overset{´}{p}}_{k}^{S,n}$ and ${\overset{´}{p}}_{k}^{S,n}$ in (P1) can be denoted in closed-form as:

(22) $$arc\;min_{p_{k}^{*C,n},p_{k}^{*S,n}}\left\{\Phi_{k}\left(p_{k}^{C,n}={\overset{´}{p}}_{k}^{C,n},p_{k}^{S,n}={\overset{´}{p}}_{k}^{S,n}\right),\Phi_{k}\left(p_{k}^{C,n}={\overset{‘}{p}}_{k}^{C,n},p_{k}^{S,n}={\overset{‘}{p}}_{k}^{S,n}\right)\right\}$$

#### 4.2.2. Joint UAV Trajectory and Frequency Association

**Figure 3:**
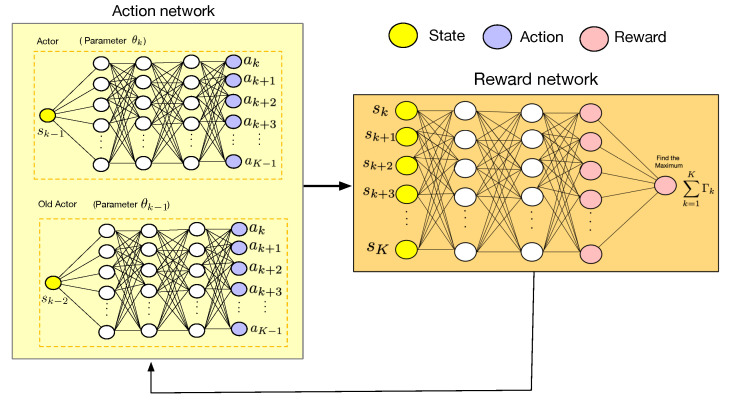
PPO algorithm.

As shown in Figure 3, we pursue an intelligent UAV trajectory optimization aided by the PPO algorithm for improving the system’s delay. The proposed PPO algorithm framework considers the UAV as a learning agent. The learning process of the PPO algorithm for the UAV by interacting with the THz environment can be expressed as:

(23) (S,A,R,γ),

where S is the state space, A is the action space, and R=S×A→R is the infinite set of rewards that contain the set of immediate rewards when moving from one state to next state resulting from the actions taken by the agents. The state, action, and reward are defined as follows:

• *State*: The states observed by an agent are determined by a combination of the transmission powers of sensing and communication. Thus, we define the state of a UAV at time step *t* as follows:
  (24) $$S_{k}^{\left(t\right)}=\left(\sum_{n=1}^{N}p_{k}^{S,n,(t)},\sum_{n=1}^{N}p_{k}^{C,n,(t)}\right).$$
• *Action*: The action is to choose proper flight direction and proper frequency association to obtain better rewards. Furthermore, we define the action performed in time-step *t* as $a_{k}^{\left(t\right)}$. Let us suppose the possibility of state $s_{k}$ taking action $a_{k}$ at time-step *t* is $P_{\theta }\left(a_{k}^{\left(t\right)}|s_{k}^{\left(t\right)}\right)$, where θ is the probability density function with parameter θ. The action is denoted by the all possible actions at time step *t*, i.e., $A_{k}^{\left(t\right)}=\left\{0∼4\pi \right\}\times f_{k,i}^{n,(t)}$.
• *Reward*: The agent receives an immediate reward, denoted as $T_{k}^{\left(t\right)}≜T\left\{s_{k}^{\left(t\right)},a_{k}^{\left(t\right)}\right\}\in R$, which describes its benefit from taking action $a_{k}^{\left(t\right)}$. Thus, the function of reward can be written as:
  (25) $$T_{n}=\sum_{k^{\prime }=k}^{K}\eta^{k^{\prime }-k}\Phi_{k},$$
  where $\eta^{k^{\prime }-k}\in \left[0,1\right]$ is the discount rate, which determines the effect of future rewards on the current action. $\eta^{k^{\prime }-k}\to 1$ means that the reward value of the future state has a great influence on the action state function, while $\eta^{k^{\prime }-k}\to 0$ means that the reward value of the future state has little influence on the action state function.

In the policy gradient algorithm, shown in Algorithm 1, the agent updates the policy by gradient augmentation. In PPO, the old actor modifies its parameters by duplicating the actor’s parameters. In order not to incur too much error, we introduce $ratio_{k}$ to limit the magnitude of rewards. In other words, when calculating the rewards, by limiting the ratio of the new policy and the old policy, the amplitudes of the state can be limited. As a result, it not only improves the stability of the PPO algorithm, but also reduces its complexity and improves the efficiency of the calculation. In this paper, the ratio of the old to new policy of each agent is calculated as follows:

(26) $$ratio_{k}=\frac{\theta_{k}}{\theta_{k-1}},\;k\in \left\{1,2,...,K\right\}.$$

Figure 3 describes the operation of the PPO algorithm. During training, a set of samples are chosen from the storage system to update the THz network parameters. The value of the network determines the choice of action through the rewards value of these sampled values. The rewards value in turn affects the sampling probability density functions. When the agent explores the THz network parameters, it will select an action at random, targeting a higher long-term reward. Furthermore, it selects the action that gains the most rewards immediately. In order to improve the sampling efficiency, PPO adopts an important sampling method to change the policy gradient algorithm from the on policy to the off policy. At this time, the update formula of the actor network is:

(27) $$\begin{array}{c} \min_{\pi_{\theta_{k}}}\Phi_{k}^{2}\left(\theta_{k}\right)=E_{s_{t}∼P_{\theta_{k}}\left(\tau \right)}\left[J_{\theta_{k}}\left(\theta_{k}\right)\right], \end{array}$$

where $\tau =\{s_{1}.a_{1},s_{2},a_{2},....,s_{K},a_{K}\}$ represents the trajectory of the agent in the entire episode.

**Figure 4:**
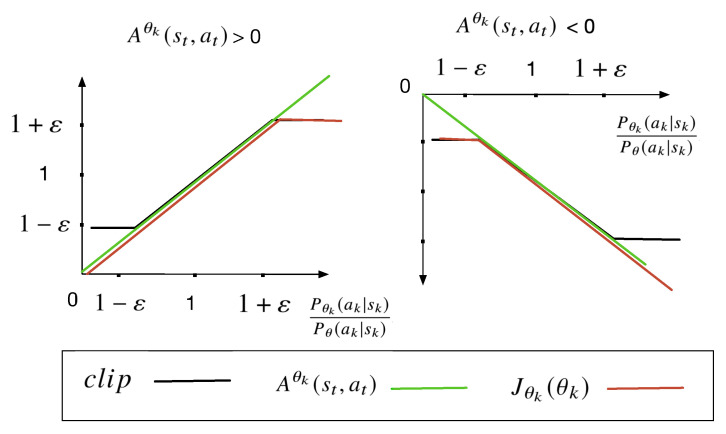
The value of $J_{\theta_{k}}\left(\theta_{k}\right)$.

PPO uses a clip function to directly limit the update range to [1−ε,1+ε]. From Figure 4, this function of PPO can be written as follows:

(28) $$\begin{array}{cc} J_{\theta_{k}}\left(\theta_{k}\right)\approx \sum_{\left(s_{t},a_{t}\right)}min\{ & \frac{P_{\theta_{k}}\left(a_{k}|s_{k}\right)}{P_{\theta }\left(a_{k}|s_{k}\right)}A^{\theta_{k}}\left(s_{t},a_{t}\right),\\ \; & clip\left(\frac{P_{\theta_{k}}\left(a_{k}|s_{k}\right)}{P_{\theta }\left(a_{k}|s_{k}\right)},1-\varepsilon ,1+\varepsilon \right)A^{\theta_{k}}\left(s_{t},a_{t}\right)\}, \end{array}$$

where ε is a hyperparameter that represents the maximum difference between $P_{\theta_{k}}$ and $P_{\theta }$. $P_{\theta_{k}}\left(\tau \right)$ interacts with the environment and $P_{\theta }\left(\tau \right)$ has already interacted with the environment. Furthermore, $A_{\theta^{k}}\left(s_{t},a_{t}\right)$ represents the estimation of the advantage function at time step *t* and can be written as:

(29) $$\begin{array}{c} A_{\theta_{k}}\left(s_{k},a_{k}\right)\approx \frac{1}{J}\sum_{j=1}^{J}\left(\Phi_{k}^{2}-E\left[\Phi_{k}^{2}\right]\right), \end{array}$$

where *J* is the number of points to sample with the probability of $P_{\theta }\left(a_{k}|s_{k}\right)$. $P_{\theta_{k}}\left(a_{k}|s_{k}\right)$ is the modified probability density function parameters ($\theta_{k}$). Furthermore, the function of clip can be written as:

$$\begin{array}{cc} \; & clip\left(x,x_{min},x_{max}\right)=\left\{\begin{array}{cc} x, & \mathrm{if}\;x_{min}\leq x\leq x_{max}.\\ x_{min}, & \mathrm{if}\;x<x_{min}.\\ x_{max}, & \mathrm{if}\;x>x_{max}. \end{array}\right. \end{array}$$

The formula for updating the action of possibility, $P_{\theta_{k}}\left(\tau \right)$, can be written as:

(30) $$\begin{array}{c} \theta_{k+1}⟵\theta_{k}+\eta^{k^{\prime }-k}▽\Phi_{k}\left(\theta_{k}\right). \end{array}$$

### 4.3. Computational Complexity

**Theorem** **2.** *The complexity of Algorithm 2 is given by $O(N+j_{max}\cdot KN)$.*

**Proof.**  In Algorithm 2, the computationally most expensive part is solving the sub-problems in (P1) (line 2) and (P2) (line 3).

In line 2 of Algorithm 2, sub-problem (P1) is solved. Every user needs to calculate function (22). Since there are *N* users, the computational complexity using method Lagrangian function is O(N).

In line 3 of Algorithm 2, sub-problem (P2) is solved by Algorithm 1. The computationally most expensive part is lines 3 and 4 of Algorithm 1. In lines 3 and 4 of Algorithm 1, we need to calculate the probability density function parameter $\theta_{k}$ and calculate the rewards function. Thus, the computational complexity is O(KN). We assume that the maximum number of iterations of Algorithm 1 is $j_{max}$. Therefore, the total computational complexity of Algorithm 1 can be written as $O(j_{max}\cdot KN)$.

To summarize, the overall computational complexity of Algorithm 2 is calculated as $O(N+j_{max}\cdot KN)$. This concludes the proof.    □

**Algorithm 1** The Proximal Policy Optimization Algorithm
1:**for** iteration = 1,2,....$j_{max}$ **do**2:    **for** action = 1,2,....K **do**3:        Run policy $\theta_{k}$ in environment for *K* time steps according to (30)4:        Compute advantage estimates $A_{\theta_{k}}$ according to (29)5:    **end for**6:    Optimize surrogate $\theta_{k}$7:    $\theta_{k+1}\leftarrow \theta_{k}$8:    Calculate $J_{\theta_{k}}\left(\theta_{k}\right)$ according to (28)9:
**end for**



**Algorithm 2** The Proposed Alternating Optimization Algorithm to Solve Problem (12)
1:**for** iteration = 1,2,....$j_{max}$ **do**2:    Solve problem (P1) for given $L_{k},f_{k,i}^{n}$ and denote the optimal solution as $P_{k}^{*C,n},P_{k}^{*S,n}$.3:    Solve problem (P2) for given $P_{k,j}^{*C,n},P_{k,j}^{*S,n}$ and denote the suboptimal solution as $L{*}_{k,j+1},f_{k,j+1}^{*n}$.4:    j = j + 1;5:
**end for**



## 5. Simulation Results

In this section, we numerically evaluate the performance of the overall alternating optimization algorithm of intelligent trajectory planning by implementing simulations in MATLAB. The radius of the UAV coverage area was set to 50 m. We set the bandwidth which is allocated to the UAV as 10 GHz. We adopted THz carrier frequencies of 300 GHz, 310 GHz, 320 GHz, 330 GHz, 340 GHz, and 350 GHz. The details of the relevant parameters are listed in Table 2.

**Figure 5:**
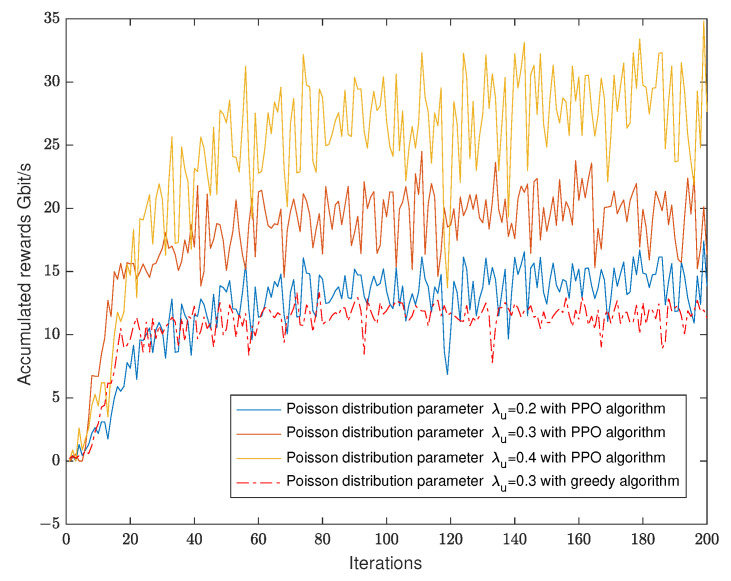
Number of iterations of the algorithms.

To investigate the convergence behavior of the proposed algorithm, we start with illustrating the accumulation of the UAV communication rate versus the number of iterations when the user Poisson distribution parameter is $\lambda_{u}=0.2,0.3$, or 0.4 persons per meter, Figure 5. It is observed that the proposed algorithm provides a higher sum rate of the system than that of the greedy sampling algorithm. This is because the PPO algorithm considers the rewards from the time of k+1 to K+1. The greedy algorithm is the result of the *k*-time obtained by mass sampling. Without considering other possible cases in general, the local optimal solution is selected each time and no backtracking is carried out, so the optimal solution is rarely obtained. This highlights the importance of the PPO algorithm, and how it theoretically gives the better sum rate for the system.

**Figure 6:**
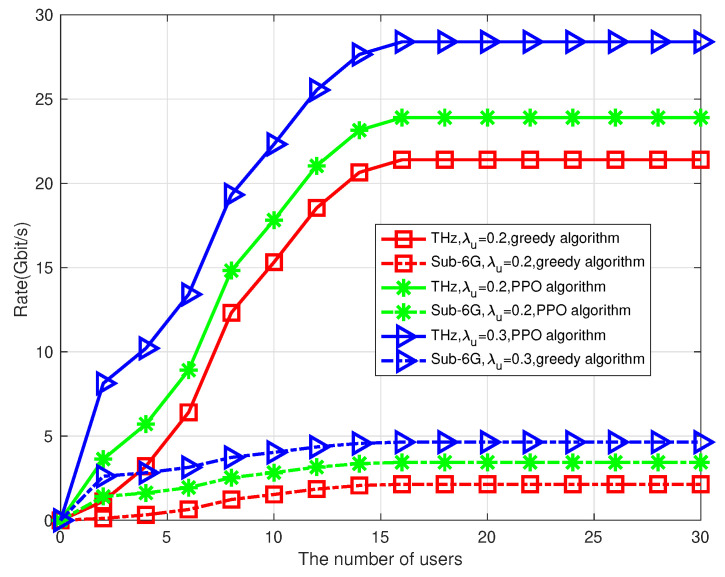
Relationship between rate and number of users.

In Figure 6, we show a comparison of the system’s sum rate in the THz and Sub-6G frequency ranges, respectively, between the proposed algorithm and the greedy algorithm under varying user distribution functions. It is discovered that the proposed algorithm provides a higher sum rate of the system than that of the greedy algorithm, because in the greedy algorithm, there is a large number of random sampling at time *k*, while the PPO algorithm not only considers the system performance at time *k*, but also considers the system performance from time *k* to time K+1. It is also discovered that the THz frequency provides a higher sum rate of the system than that of the Sub-6G. That is because the signal-to-noise ratio is much higher at the terahertz frequency than at the sub-6G frequency due to the high pathloss characteristic of THz channel resulting in low interference between users. This highlights the importance of an appropriate algorithm for the THz frequency.

**Figure 7:**
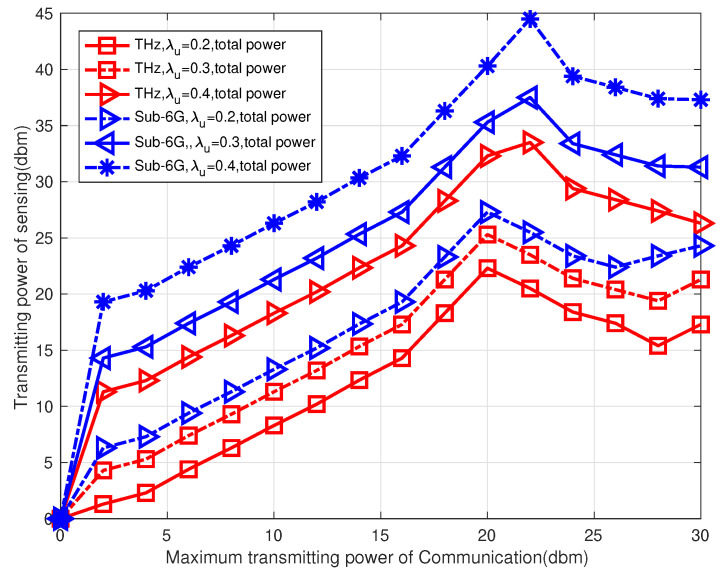
Relationship between sensing and communication powers.

In Figure 7, the relationship between the maximum communication power and sensing power is shown. It is observed that as the maximum communication power increases, the transmitting sensing power increases, but once the maximum value is reached, the sensing and communication powers start to decrease to maintain the same communication rate. This is because the communication and sensing signals share a spectrum. When the value of the maximum transmitted communication power increases, the THz-UAV increases the power of communication in order to obtain a higher information rate. As a result of the C5 constraint, the sensing power becomes smaller. The precision of sensing the terahertz channel will be affected by the decrease in sensing power. This will affect the allocation of the THz-UAV channel and cause the information rate to decrease. Therefore, there must be a maximum value of the sensing power to obtain the minimum delay.

**Figure 8:**
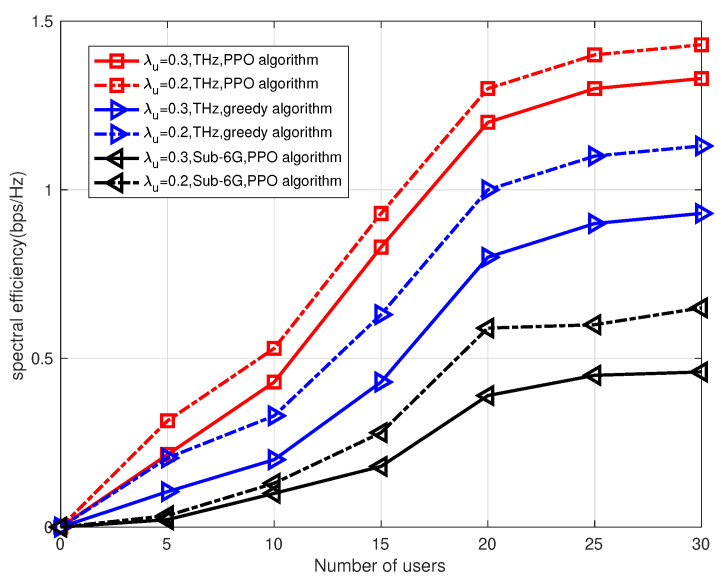
Relationship between frequency efficiency and number of users.

In Figure 8, we show the relationship between frequency efficiency in the THz and Sub-6G frequencies, respectively. The numbers of users under the parameter of user density function are $\lambda_{u}=0.2$ and $\lambda_{u}=0.3$. We can see that as the number of users increases, the frequency efficiency increases. This is due to the fact that as the number of users increases, the information rate has been greatly improved. As can be seen from the figure, with the same number of users, the higher the user density function parameter, the lower the spectrum density. This is because when the user density function parameter is higher, the interference between users is stronger, resulting in a reduction in the information rate, so the spectral efficiency is lower. Therefore, the frequency spectrum efficiency of THz wireless communication is easily affected by the user density.

## 6. Conclusions

This paper investigated the problem of joint UAV trajectory, frequency association, and power optimization, aiming to minimize the sum delay in the terahertz band. The sum delay minimization was formulated as a convex optimization problem. This problem was transformed into the Lagrange multiplier method and a PPO problem. A Lagrange sub-problem was devised, aiming to obtain the sensing and communication powers. A PPO algorithm was devised to obtain the UAV trajectory and frequency association. Our results showed that the proposed algorithm achieved a good performance with a significant increase in the sum delay compared with the greedy algorithm and the Sub-6G frequency scenario, indicating its potential in a practical design. However, the method used in this paper has not used in a real UAV. Thus, there is a certain gap between theory and practice, which provides a direction for future research.

## Figures and Tables

**Table 1:** Our novel contribution contrasted to the state-of-the-art in UAV communication research.

	[12,13,14,15,16]	[17,18,19]	[20,21,22]	[23,24,25,26]	Our Work
THz Frequency	×	✔	×	✔	✔
UAVs Communication	✔	✔	×	×	✔
Power Optimization	✔	×	×	✔	✔
Integrated Sensing and Communication	×	×	✔	×	✔
UAV Trajectory Design	✔	✔	×	✔	✔

**Table 2:** Simulation parameters.

Parameter	Value	Parameter	Value
Time, *T*	20 s	A-BS Height, *H*	5 m
Time slot, K+1	1 ms	ABS Speed, *V*	[0,3] m/s
Noise power, $N_{0}$	−20 bBm	Reference pressure, $p_{0}$	101.325 kPa
Reference temperature, $T_{STP}$	20	Maximum sensing transmission power, $p_{k}^{S,n}$	30 dBm
Maximum communication transmission power, $p_{k}^{C,n}$	30 dBm	Discount rate, η	0.1

## Data Availability

The data presented in this study are available on request from the corresponding author. The data are not publicly available due to legal restrictions.

## Appendix A

**Proof.** The second-order derivative of objective function (13) with respect to $p_{k}^{C,n}$ and $p_{k}^{S,n}$ can be, respectively, obtained by

(A1) $$\frac{\partial^{2}\Phi_{k}^{1}}{\partial^{2}p_{k}^{C,n}}=\sum_{n=1}^{N}2\frac{D_{n}}{B_{u}ln2}\lambda_{1}\frac{1}{{\left(1+\gamma_{k}^{n}\right)}^{2}}\left(\lambda_{1}-\frac{1}{log(1+\gamma_{k}^{n})}\right)\geq 0$$

where $\lambda_{1}=\frac{h_{k}^{n}\left(f_{k,i}^{n}\right)}{N_{0}+p_{k}^{S,n}h_{k}^{n}\left(f_{k,i}^{n}\right)-p_{k-1}^{S,n}h_{k-1}^{n}\left(f_{k,i}^{n}\right)+\sum_{j=1}^{j/n}p_{k}^{C,j}h_{k}^{j}\left(f_{k,i}^{j}\right)+p_{k}^{S,j}h_{k}^{j}\left(f_{k,i}^{n}\right)-p_{k-1}^{S,j}h_{k-1}^{j}\left(f_{k,i}^{n}\right)}$.

(A2) $$\frac{\partial^{2}\Phi_{k}^{1}}{\partial^{2}p_{k}^{S,n}}=\sum_{n=1}^{N}2\frac{D_{n}}{B_{u}ln2}\lambda_{2}\frac{1}{{\left(1+\gamma_{k}^{n}\right)}^{2}}{\left(\frac{h_{n}^{n}\left(f_{k,i}^{n}\right)+\lambda_{2}}{p_{k}^{C,n}h_{n}^{n}\left(f_{k,i}^{n}\right)}\right)}^{2}\left(2+r_{k}^{n}+\frac{h_{n}^{n}\left(f_{k,i}^{n}\right)+\lambda_{2}}{p_{k}^{C,n}h_{n}^{n}\left(f_{k,i}^{n}\right)}\right)\geq 0$$

where $\lambda_{2}=N_{0}-p_{k-1}^{S,n}h_{k-1}^{n}\left(f_{k,i}^{n}\right)+\sum_{j=1}^{j/n}p_{k}^{C,j}h_{k}^{j}\left(f_{k,i}^{j}\right)+p_{k}^{S,j}h_{k}^{j}\left(f_{k,i}^{n}\right)-p_{k-1}^{S,j}h_{k-1}^{j}\left(f_{k,i}^{n}\right)$. Therefore, the problem (P1) is convex. This concludes the proof. □
